# The Dynamic Changes in the Main Substances in *Codonopsis pilosula* Root Provide Insights into the Carbon Flux between Primary and Secondary Metabolism during Different Growth Stages

**DOI:** 10.3390/metabo13030456

**Published:** 2023-03-21

**Authors:** Sheng-Song Wang, Tong Zhang, Long Wang, Shuai Dong, Dong-Hao Wang, Bin Li, Xiao-Yan Cao

**Affiliations:** 1 Key Laboratory of the Ministry of Education for Medicinal Resources and Natural Pharmaceutical Chemistry, National Engineering Laboratory for Resource Development of Endangered Crude Drugs in Northwest of China, Shaanxi Normal University, Xi’an 710062, China; 2 Institute of Botany of Shaanxi Province, Xi’an Botanical Garden of Shaanxi Province, Xi’an 710061, China

**Keywords:** *Codonopsis pilosula*, atractylenolide III, polysaccharides, carbon flux, gene expression, secondary metabolites

## Abstract

The dried root of *Codonopsis pilosula* (Franch.) Nannf., referred to as Dangshen in Chinese, is a famous traditional Chinese medicine. Polysaccharides, lobetyolin, and atractylenolide III are the major bioactive components contributing to its medicinal properties. Here, we investigated the dynamic changes of the main substances in annual Dangshen harvested at 12 time points from 20 May to 20 November 2020 (from early summer to early winter). Although the root biomass increased continuously, the crude polysaccharides content increased and then declined as the temperature fell, and so did the content of soluble proteins. However, the content of total phenolics and flavonoids showed an opposite trend, indicating that the carbon flux was changed between primary metabolism and secondary metabolism as the temperature and growth stages changed. The changes in the contents of lobetyolin and atractylenolide III indicated that autumn might be a suitable harvest time for Dangshen. The antioxidant capacity in Dangshen might be correlated with vitamin C. Furthermore, we analyzed the expression profiles of a few enzyme genes involved in the polysaccharide biosynthesis pathways at different growth stages, showing that *CpUGpase* and CPPs exhibited a highly positive correlation. These results might lay a foundation for choosing cultivars using gene expression levels as markers.

## 1. Introduction

*Codonopsis pilosula* (Franch.) Nannf., which belongs to the Capamnulaceae family, is mainly distributed in East Asia and especially concentrated in China [1]. Its dried root (referred to as Dangshen in Chinese) is mainly employed in traditional Chinese medicine to replenish qi (vital energy), strengthen the spleen, tone the lungs, and nourish the blood [2], and has been used extensively since the *Qing* dynasty [3]. Modern research has shown that Dangshen contains various bioactive constituents, including polysaccharides, alkaloids, polyacetylenes, terpenoids, flavonoids, phenolic acids, volatile oils, and so on [4]. Among these compounds, *C*. *pilosula* polysaccharides (CPPs) have been proven to possess multiple pharmacological activities, including antioxidation, anti-aging, anti-tumor, anti-diabetic, and immune-enhancing functions [4,5,6]. Lobetyolin, which is a polyacetylene glycoside, has been suggested to be the index compound of Dangshen, as it might serve as a potential bioactive marker related to hematopoietic and immunologic functions [7]. Furthermore, lobetyolin has anticancer, antioxidant, and cardioprotective activities [8]. Atractylenolide III (a terpenoid) is a potent anti-inflammatory and anti-cancer agent that may be useful in treating asthma [9,10]. Phenolic compounds, carotenoids, and vitamin C are associated with antioxidant activity [11]. Dangshen is also a popular dietary supplement when cooking porridge, making soup or tea, and producing wine. In 2019, it was included in the list of food and traditional Chinese medicine substances by the National Health Commission of the People’s Republic of China. (www.nhc.gov.cn/sps/s7885/202001/1ec2cca04146450d9b14acc2499d854f.shtml, accessed on 6 January 2020). Due to its significant medicinal and health-promoting value, the demand for Dangshen is increasing [12].

As a medicine, the quality of Dangshen is determined by the contents of bioactive primary and secondary metabolites. The diversion of carbon flux between these metabolites leads to a balance between growth and defense throughout a plant’s life [13]. Generally, growth is related to primary metabolites, such as polysaccharides and soluble proteins, whereas defense depends on secondary metabolites, including phenolic acids, flavonoids, etc. Recent studies have indicated that the contents of bioactive compounds in Dangshen are influenced by geographic regions, cultivation methods, and processing techniques [14]. Furthermore, the application of methyl jasmonate (MeJA) can promote the accumulation of bioactive metabolites in *C. pilosula* by regulating the expression of phosphomevalonate kinase (*CpPMK*) and UDP-glucose pyrophosphorylase (*CpUGPase*) enzyme genes involved in the biosynthesis of terpenoids and carbohydrates [15]. It has been acknowledged that the partitioning of carbon resources between growth and defense is impacted by environmental factors such as temperature [16]. Although the dynamic changes of a few active ingredients in Dangshen that occur during different harvesting periods have been investigated [17], the carbon flux between primary and secondary metabolism influenced by environmental factors remains unclear. Thus, the metabolic changes during different developmental stages deserve further comprehensive investigation.

The present study investigated the dynamic changes of the main active compounds in the roots of annual *C. pilosula* during different growth stages (12 time points from 20 May, early summer, to 20 November, early winter, 2020). Simultaneously, the expression profiles of a few enzyme genes involved in their biosynthetic pathways during different growth stages were determined with correlation analysis. Our results indicated that the carbon flux between primary and secondary metabolism in Dangshen could be influenced by temperature during different growth stages. In addition, our study demonstrated that autumn was the most suitable harvest season for Dangshen, which was a significant factor for the quality and yield. Furthermore, a few enzyme genes involved in the polysaccharide biosynthesis pathways could be used as biomarkers for cultivar selection.

## 2. Materials and Methods

### 2.1. Chemicals and Reagents

Standard compounds, such as lobetyolin, atractylenolide III, β-carotene and several types of monosaccharides (D-mannose, rhamnose, D-glucuronic acid, D-galacturonic acid, glucose, galactose, and arabinose), and vitamins (vitamin B3, vitamin B6, inositol, and vitamin C), were purchased from the National Institutes for Food and Drug Control (Beijing, China). 1,1-diphenyl-2-picrylhydrazyl (DPPH) and 2,2-Azino-bis (3-ethylbenzothiazoline-6-sulphonic acid) diammonium (ABTS) were produced by Solarbi (Beijing, China). Acetonitrile and methanol were LC-MS grade and obtained from Thermo Scientific, Waltham, United States. 1-phenyl-3-methyl-5-pyrazolon (PMP) was obtained from Sigma-Aldrich, United States.

### 2.2. Plant Materials

The seeds of *Codonopsis pilosula* (Franch.) Nannf. were bought from farmers in Longxi County, Gansu Province, China, and identified by Professor Zhezhi Wang. Partial seeds were sown in the same place in May 2019 (Figure S1), while the rest were stored at the National Engineering Laboratory for Resource Development of Endangered Crude Drugs in Northwest China of Shaanxi Normal University. The *C. pilosula* roots were collected every two weeks from 20 May to 20 November 2020 (early summer to early winter; 12 times in total and 30 samples for each collection). All collected samples (2.1351 g, 3.3872 g, 21.2493 g, 21.6647 g, 29.0010 g, 33.7751 g, 43.5779 g, 52.6055 g, 57.7832 g, 61.3328 g, 60.4109 g, and 81.8263 g for each collection) were separated equally into two portions. One portion was flash-frozen with liquid nitrogen and stored at −80 °C, pending RNA extraction [18]. The other portion was air-dried in the shade, pulverized to a powder, and then sifted using a 40-mesh sieve, which was employed for the quantitative analysis of the metabolites [19].

### 2.3. Determination of Crude Polysaccharide Content and Monosaccharide Composition Assay

The polysaccharide was extracted according to the published literature with slight modifications [20]. Briefly, 0.2 g powder was defatted with 4 mL absolute ethanol under stirring for 20 h at room temperature, followed by centrifugation (4000 rpm, 15 min), after which the defatted precipitate was collected and dried for extraction of C. pilosula polysaccharides. The precipitate was dissolved in deionized water at a material-to-liquid ratio of 1:30 (g/mL) and left at 63 °C for 1.25 h. The solution was filtered, and the residue was extracted two additional times. The filtrate was concentrated to 20% of the original volume under reduced pressure, and the Sevag method [21] was used to remove the protein. The polysaccharides were precipitated by slowly adding three volumes of anhydrous alcohol and stored at 4 °C overnight. Subsequently, the precipitate was freeze-dried to obtain the crude CPPs. The yield of the crude CPPs was calculated using the following equation: CPP yield (%) = weight of CPPs (g)/weight of raw material (g) [20].

The hydrolysis of the crude CPPs and the PMP derivatization of the monosaccharides was performed according to a published method [20]. The analysis of PMP-labeled monosaccharides was conducted using an UltiMate™ 3000HPLC instrument (Thermo, USA) equipped with a ZORBAX Eclipse XDB-C18 column (250 mm × 4.6 mm, 5 μm, Agilent, Santa Clara, CA, USA). The wavelength of the diode array detector (DAD) was 250 nm. Elution was carried out at a 1.0 mL/min flow rate at 30 °C. Mobile phase A consisted of a 0.5 M phosphate buffer solution (PBS) (pH 6.8), whereas mobile phase B comprised acetonitrile. The ratio of the mobile phase was 4:1 (A:B). The injection volume was 10 μL [20].

### 2.4. Soluble Protein Analysis

The soluble protein content of Dangshen was quantified by the Coomassie blue method. The dye reagent was incubated with 0.1 mL of the sample for 4 min at room temperature. The A_595_ of the dye–protein sample minus that of a blank solution of 0.1 mL water with reagent was measured [22].

### 2.5. Total Phenolics, Total Flavonoids, and Anthocyanins Assay

The total phenolic, flavonoid, and anthocyanin levels of the Dangshen were quantified by our laboratory procedures [23]. For total phenolics and flavonoids, 0.2 g of powder was extracted with 6 mL of acidified (0.3% HCl, *v*/*v*) methanol for 24 h at 4 °C. Then, the samples were centrifuged at 4 °C for 30 min at 5000× *g*. The pellet was washed with 2 × 15 mL of solvent. Supernatants were combined to obtain the total extract and stored at 4 °C. Total phenolics were measured using the Folin–Ciocalteu method [24]. Total flavonoids were determined following the Dewanto procedure [25].

For anthocyanins, after centrifugation at 21,500× *g* for 3 min at room temperature, 0.4 mL of the supernatant was added to 0.6 mL of acidic methanol. Absorption at 530 nm and 657 nm was determined and used to quantify anthocyanins with the following equation: concentration of anthocyanins = (A_530_ − 0.25 A_657_)/weight [26].

### 2.6. Determination of Lobetyolin and Atractylenolide III

A 0.5 g sample of root powder was extracted with 50 mL absolute ethanol in an ultrasonic bath for 45 min at 40 °C. The extracted solutions were filtered, and the filtrate was freeze-dried and dissolved in 5 mL methanol. One microgram of lobetyolin or atractylenolide III was dissolved in 5 mL methanol as the standard solution. The quantitative detection of lobetyolin and atractylenolide III was conducted using an UltiMate™ 3000 HPLC instrument (Thermo, USA) equipped with ZORBAX Eclipse XDB-C18 column (250 mm × 4.6 mm, 5 μm, Agilent, USA). The gradient elution consisted of 0.1 mol/L PBS (A) and acetonitrile (B). The gradient profile was as follows: 0–25 min, 10–40% B; 25–30 min, 40–80% B; 30–35 min, 80% B; 35–40 min, 80–100% B; and 40–42 min, 100–10% B. Lobetyolin and atractylenolide III were detected at 220 nm at a 1.0 mL/min flow rate at 30 °C [27].

### 2.7. Determination of Vitamins and β-Carotene

To determine the Dangshen vitamin content, the dried powder (0.3 g) was extracted with 0.1 mol/L hydrochloric acid in an ultrasonic bath for 30 min, followed by a boiling water bath for 30 min. The supernatant was collected via a 0.22 μm pore filter membrane and then used for HPLC analysis. The mobile phase comprised 0.1% phosphoric acid (A) and methanol (B). Isocratic elution was performed with 15% B. Vitamin C was detected at 272 nm at a 1.0 mL/min flow rate at 25 °C [28].

To determine the content of β-carotene, the dried powder (0.3 g) was immersed in a 5 mL extracting solution (petroleum ether/acetone = 8/2) in an ultrasonic bath for 15 min and subsequently centrifuged for 10 min. This process was repeated several times until the extracted solution was colorless [29], which was filtered using a 0.22 μm pore filter membrane for further HPLC analysis. The mobile phase comprised 90% acetonitrile and 10% methanol (9:1, *v*/*v*). The β-carotene was detected by a DAD at 450 nm at a flow rate of 1.0 mL/min at 25 °C [29].

### 2.8. Antioxidant Capacity Assay

The scavenging activities of 1,1-diphenyl-2-picrylhydrazyl (DPPH) and 2,2-Azino-bis (3-ethylbenzothiazoline-6-sulphonic acid) diammonium (ABTS) radicals were measured as described in a previous study [30]. Briefly, 0.05 g powder was extracted with 1 mL 70% ethanol in an ultrasonic bath for 30 min at 40 °C, followed by centrifugation (10,000 rpm, 10 min), and the supernatant was used as the sample. For the analysis of DPPH radical scavengers, a 100 μL sample solution was mixed with 2 mL of a 0.2 mmol·L^−1^ DPPH ethanol solution. After 30 min of incubation in the dark at room temperature, the absorbance of the mixture (*A*_1_) and the blank control (*A*_0_, 70% ethanol instead of the sample solution) was measured at 515 nm on a Spectral photometer machine. The scavenging activity was calculated using the formula (*A*_0_ − *A*_1_)/*A*_0_.

To analyze ABTS radical scavengers, 7.4 mM ABTS and 2.6 mM Na_2_S_2_O_8_ were mixed at a volume ratio of 1:1 and reacted overnight in the dark at 37 °C. The solution was then diluted with phosphate-buffered saline (pH 7.0) to an absorbance of 0.70 ± 0.02 at 734 nm to obtain the ABTS working solution. A 100 μL sample solution was mixed with 2 mL of the ABTS working solution and incubated at room temperature for 10 min, followed by the determination of the absorbance of the mixture (*A*_2_) and blank control (*A*_0_, 70% ethanol instead of sample solution) at 734 nm. The scavenging activity was calculated using the formula (*A*_0_ − *A*_2_)/*A*_0_.

### 2.9. Expression Pattern of Genes Involved in Primary and Secondary Metabolism (Carbohydrate and Terpenoid Biosynthesis Pathways)

The total RNA of the *C. pilosula* roots at different developmental stages was extracted with a Quick RNA Isolation Kit (Huayueyang, Beijing, China) and then reversed to cDNA with the TransScript II First-Strand cDNA Synthesis SuperMix (TransGen Biotech, Beijing, China) [31]. The cDNAs were employed as templates to analyze the relative expression levels of several enzyme genes by qRT-PCR, and *CpGAPDH* was used as the internal gene [31]. All qRT-PCR reactions were performed using SYBR Green Master Mix (TaKaRa, Japan) on a Roche lightcycle96 real-time machine with the following programs: 95 °C for 30 s, then 40 cycles of 95 °C for 10 s and 60 °C for 20 s. Each qRT-PCR experiment had three biological and three technical replicates [31]. All of the primers are listed in Table S1.

### 2.10. Statistical Analysis of the Data

The data are shown as means ± SE (standard error) of three replicates. The statistical analysis of the data was performed using SPSS Version 19.0 (SPSS Inc. Chicago, IL, USA). Different letters above the bars indicate a significant difference (*p* < 0.05) by one-way ANOVA with Tukey’s multiple comparisons test. Pearson’s correlation was used to identify the relationship between the active ingredients and marker genes. Correlation heat maps were generated using the HIPLOT website (https://hiplot.com.cn/basic/cor-heatmap, accessed on 31 December 2022).

## 3. Results

### 3.1. Root Biomass at Different Development Stages Showed a Continuous Increase

**Figure 1:**
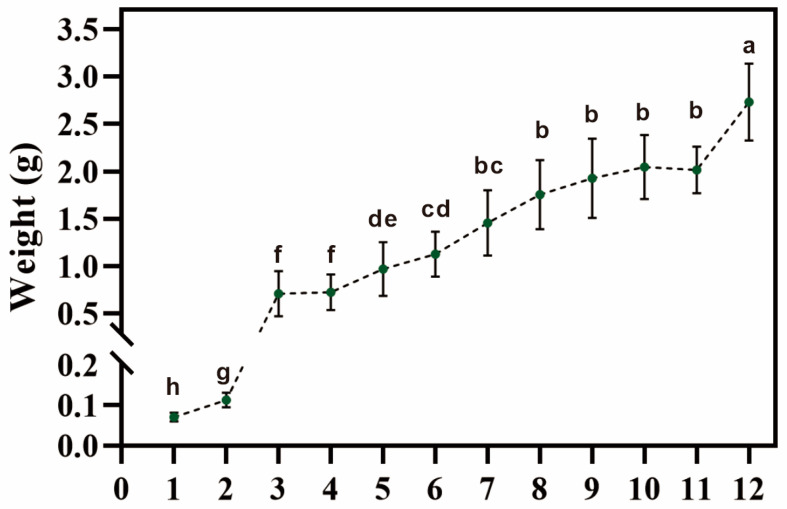
Root biomass at different development stages. Dry weights of each root at different developmental stages. More than 30 samples were measured at each time point (*n* ≥ 30). Numbers 1–12 on the x-axis represent the 12 time points, which were 20 May, 5 June, 20 July, 20 August, 5 September, 20 September, 5 October, 20 October, 5 November, and 20 November. Different letters above the bars indicate a significant difference (*p* < 0.05) by one-way ANOVA with Tukey’s post-hoc test.

Since the above-ground part of *C. pilosula* sprouted in May and withered in November, a total of 12 time points were designed during that period. The morphologies of the roots were observed and statistically analyzed. As seen in Figure S2, the lengths and diameters of the taproots gradually increased, and a few fine fibrous roots were formed. The dry weight of the *C*. *pilosula* root increased sharply from 5 June to 5 July, with a 6.45-fold change consisting of an increase from 0.11 to 0.71 g. This gradually increased until 20 October, followed by a significant increase from 5 to 20 November, with the highest weight of 2.73 g on 20 November (Figure 1).

### 3.2. Quantitative Analysis of Polysaccharides, Soluble Protein, and Total Phenolics and Flavonoids Indicated the Carbon Flux between Primary and Secondary Metabolism in Dangshen

**Figure 2:**
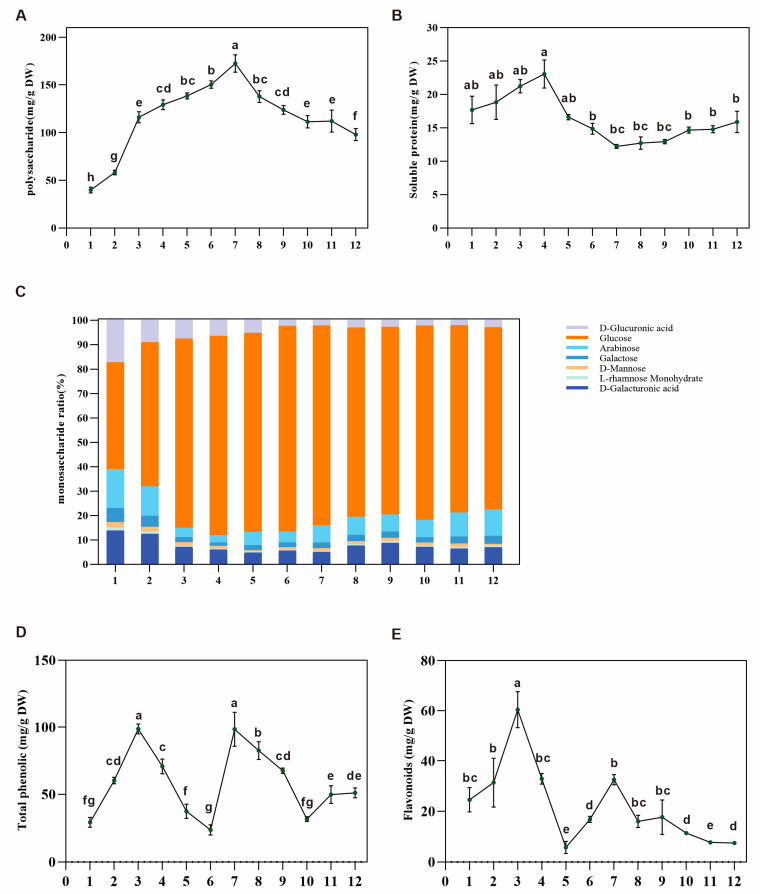
Crude polysaccharide, soluble protein, total phenolic acid and flavonoid contents, and the ratio of seven monosaccharides in the root of *Codonopsis pilosula* at different growth stages. (**A**) Dynamic changes in crude polysaccharide content during these periods. (**B**) Dynamic changes in soluble protein content during these periods. (**C**) The ratio of seven monosaccharides in the crude polysaccharides of Dangshen. (**D**) Dynamic changes in total phenolic acid content during these periods. (**E**) Dynamic changes in flavonoid content during these periods. DW: dry weight. Values represent means ± SE (*n* ≥ 3). Different letters above the bars indicate a significant difference (*p* < 0.05) by one-way ANOVA with Tukey’s multiple comparisons test.

Polysaccharides are an essential active ingredient of Dangshen. We determined the content of crude polysaccharides, which showed an upward trend from 20 May to 20 September with the highest level of 172.50 mg/g DW, which then declined slowly and dropped to 97.99 mg/g DW after the temperature fell (Figure 2A). The monosaccharide composition of the crude CPPs was confirmed by HPLC (Figure S3). Among the seven monosaccharides (mannose, rhamnose, glucuronic acid, galacturonic acid, glucose, galactose, and arabinose), glucose, as the primary source of energy and the typical form of carbohydrate intake in organisms, accounted for the dominant proportion and ranged from 43.67 to 84.19% during different growth stages (Figure 2C). Meanwhile, the proportions of other monosaccharides showed different change patterns. The proportion of glucuronic acid gradually decreased from 17.32% and stabilized at ~2.5% on 5 August, whereas that of mannose, galacturonic acid, and galactose showed a downward trend.

Soluble protein is an important appraisal index for assessing plants’ nutritional value and quality. The soluble protein content in Dangshen varied from 12.20 to 23.05 mg/g DW during different growth stages (Figure 2B). It increased to the highest level on 20 July, then gradually decreased when the temperature began to fall.

Phenolics and flavonoids usually play critical roles in regulating plant growth and metabolism, responding to stress, etc. We found that the contents of total phenolics and flavonoids varied from 23.69 to 98.69 mg/g DW and 5.79 to 60.34 mg/g DW, respectively (Figure 2D,E). We found that the total phenolic content initially increased with the accumulation of root biomass but decreased at the stage of high biomass accumulation when polysaccharide content increased rapidly. This result indicated that the carbon flux changed between primary and secondary metabolism as the temperature and growth stages changed.

### 3.3. The Contents of Lobetyolin and Atractylenolide III Might Provide Information for the Harvest Time of Dangshen

**Figure 3:**
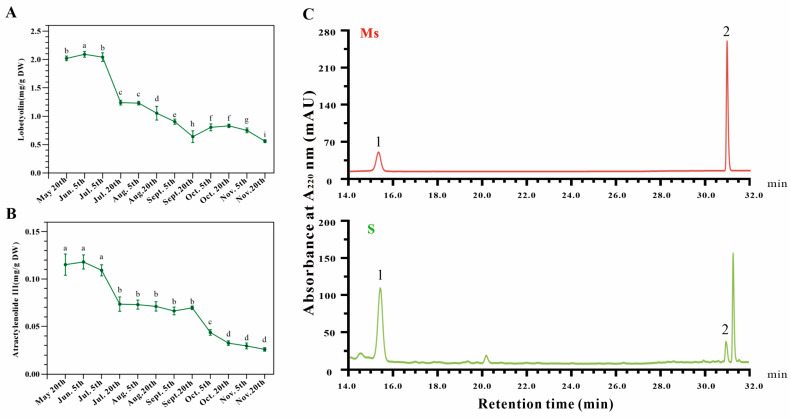
Dynamic changes in the lobetyolin (**A**) and atractylenolide III (**B**) contents of *Codonopsis pilosula* root at different growth stages. Values represent means ± SE (*n* ≥ 3). DW: dry weight. Different letters above the bars indicate a significant difference (*p* < 0.05) by one-way ANOVA with Tukey’s multiple comparison test. (**C**) HPLC profiles of lobetyolin and atractylenolide III. 1, lobetyolin; 2, atractylenolide III. Ms: mixed standards; S: Sample.

As the general marker compounds of Dangshen, the levels of lobetyolin and atractylenolide III were investigated by HPLC. The contents of lobetyolin and atractylenolide III ranged from 2.090 to 0.559 mg/g DW and 0.115 to 0.027 mg/g DW, respectively (Figure 3). It is worth noting that these two compounds exhibited similar profiles during the various growth periods. They were stable and exhibited a higher level from 20 May to 5 July, then significantly decreased on 20 July and remained stable until 20 September. The level of atractylenolide III significantly declined from 20 September to 5 October. Meanwhile, the lobetyolin content showed a slight rebound after September, though it decreased to 0.559 mg/g DW at the last time point. Given this, the most suitable harvest time of Dangshen is autumn, which was consistent with the change patterns of glucose and soluble protein.

### 3.4. Antioxidant Capacity in Dangshen Was Correlated with Vitamin C Content

**Figure 4:**
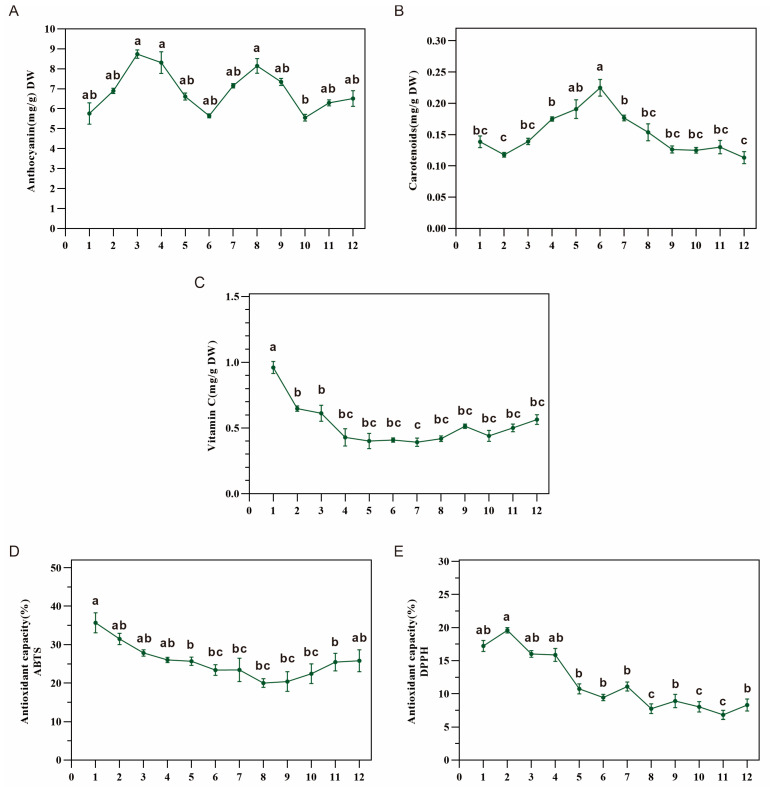
The changes in the contents of anthocyanin (**A**), β-carotene (**B**), and vitamins C (**C**), and in the scavenging abilities of ABTS (**D**) and DPPH (**E**) radicals in the root of *C. pilosula* during its various growth stages. Values represent means ± SE (*n* ≥ 3). DW: dry weight. Different letters above the bars indicate a significant difference (*p* < 0.05) by one-way ANOVA with Tukey’s multiple comparison test.

Many bioactive compounds, including vitamin C, anthocyanins, and β-carotene, contribute to antioxidant activities. The anthocyanin levels fluctuated from 5.48 to 9.18 mg/g DW and exhibited similar change patterns for the two periods from 20 May to 20 August and from 20 August to 20 October (Figure 4A). The β-carotene content gradually increased and attained its highest point of 0.22 mg/100 g on 20 August, followed by an incremental decrease (Figure 4B). The content of vitamin C decreased significantly in the early stage. Then, it remained at a stable level (Figure 4C).

Measurement of the scavenging abilities of DPPH and ABTS radicals is a general method for verifying their antioxidant capacities. Our results indicated that their antioxidant capacities decreased with root growth, and the scavenging ability of the DPPH radicals exhibited a similar trend to that of the ABTS radicals (Figure 4D,E). The total antioxidant capacity of Dangshen had a similar trend in terms of the vitamin C content, indicating that the antioxidant capacity in Dangshen might be correlated with vitamin C.

### 3.5. Correlation Analysis of Carbohydrate, Atractylenolide III, and Expression of Related Genes 

**Figure 5:**
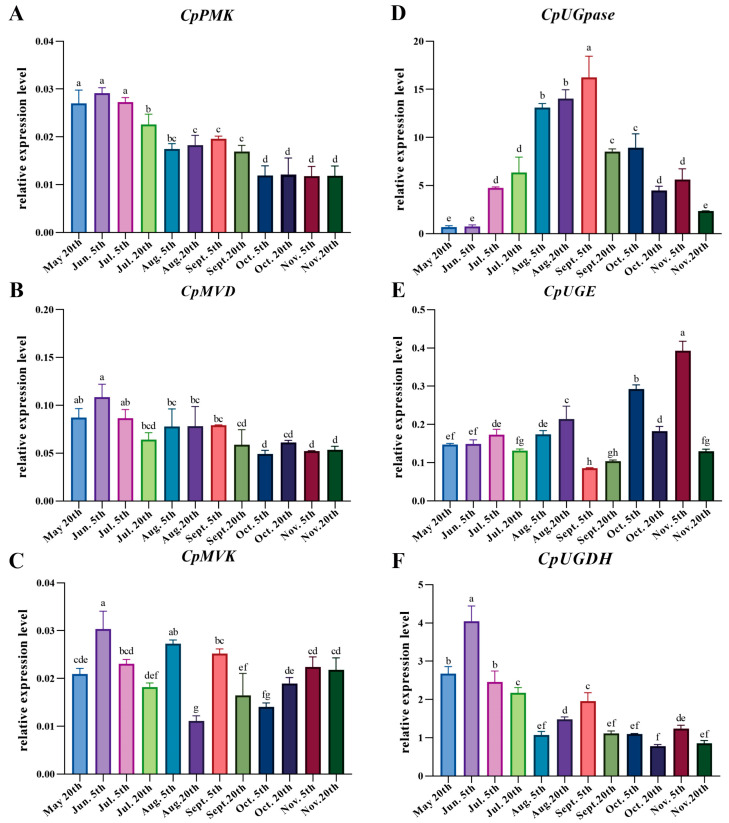
Expression patterns of the key enzyme genes involved in the terpenoid and carbohydrate biosynthetic pathways during the different growth stages determined by qRT-PCR. All data represent means ± SE (*n* ≥ 3). Different letters above the bars indicate a significant difference (*p* < 0.05) by one-way ANOVA with Tukey’s multiple comparison test. (**A**) The relative expression of *CpPMK*. (**B**) The relative expression of *CpMVD*. (**C**) The relative expression of *CpMVK*. (**D**) The relative expression of *CpUGpase*. (**E**) The relative expression of *CpUGE*. (**F**) The relative expression of *CpUGDH*.

To investigate the molecular mechanisms of the fluctuations of active compounds during different growth stages, the expression levels of a few candidate genes involved in the terpenoid (*CpPMK*, *CpMVK*, and *CpMVD*) and carbohydrate (*CpUGpase*, *CpUGE*, and *CpUGDH*) biosynthesis pathways were determined. As shown in Figure 5, the expression profiles of these genes were different. *CpPMK* was expressed at a higher level in the early stage, its expression then declined between 20 July and 5 August, and it then remained stable at a lower level from 5 October to 20 November (Figure 5A), which was similar to the patterns of atractylenolide III accumulation. Although the pattern of change in *CpMVD* was similar to that of *CpPMK*, the variation range of *CpMVD* was smaller than that of *CpPMK* for the 12 time points (Figure 5B). *CpMVK* showed the highest and lowest expression levels on 5 June and 20 August, respectively (Figure 5C). The expression level of *CpUGpase* was significantly lower on 20 May and 5 June, which swiftly increased to a peak on 5 September, followed by a declining trend (Figure 5D), which was similar to the pattern of change in polysaccharides during the different growth stages. *CpUGE* was relatively stable during the first five time points and showed the highest expression level on 5 November (Figure 5E), while the expression of *CpUGDH* showed almost the opposite pattern of change relative to that of *CpUGE* (Figure 5F), which was consistent with the change in glucuronic acid contents during the different growth stages.

**Figure 6:**
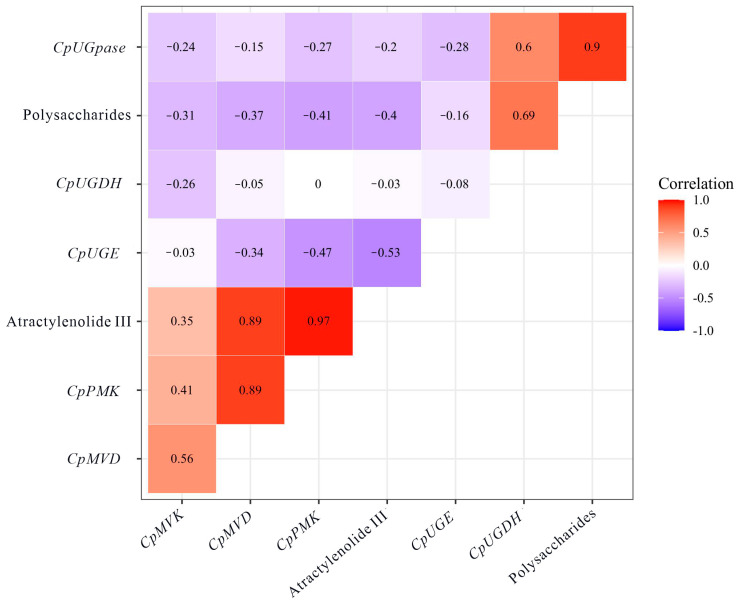
Pearson’s correlation matrix for multiple genes, atractylenolide III, and *Codonopsis pilosula* polysaccharides. The scale on the right side shows color codes with corresponding r values. Different letters above the bars indicate a significant difference (*p* < 0.05) by one-way ANOVA with Tukey’s post-hoc test.

We performed a correlation analysis of the contents of CPPs, atractylenolide III, and the expression levels of six genes. The results indicated that the content of atractylenolide III was positively correlated with the expression levels of *CpPMK* (*R*^2^ = 0.97) and *CpMVD* (*R*^2^ = 0.89). Furthermore, *CpUGpase* and CPPs exhibited a highly positive correlation (*R*^2^ = 0.90) (Figure 6).

## 4. Discussion

Dangshen possesses excellent edible and medicinal value and has been widely used for centuries [3]. In Chinese Pharmacopoeia, the dried roots of three species of *Codonopsis*, including *C pilosula*, *C*. *pilosula* Nannf. var. *modesta* (Nannf.) L.T. Shen, and *C*. *tangshen*, are officially listed under the name Radix Codonopsis or Dangshen. It was discovered that the contents of both lobetyolin and atractylenolide III are higher in *C. pilosula* than in the other two species [19]. Due to reckless mining, wild Dangshen resources have been practically exhausted; thus, the Dangshen sold at the market is mostly artificially cultivated. Studies have shown that multiple factors contribute to the content of active components in Dangshen, such as the cultivated species, geographic regions, cultivation methods, harvest periods, and processing techniques [14,19]. Although Dangshen is traditionally harvested in autumn, little is known about the dynamic changes in metabolites during its different growth stages. In the present study, the dynamic changes of some primary metabolites (e.g., crude polysaccharides and protein) and secondary metabolites (vitamin C, β-carotene, lobetyolin, atractylenolide III, anthocyanins, total phenolics, and total flavonoids) in the dried roots of annual *C. pilosula* from 20 May to 20 November 2020 were investigated. Additionally, the expression levels of several genes involved in CPPs at different growth stages were analyzed. The results provided the theoretical basis for rational C. *pilosula* development and utilization.

As the main active substances of water-soluble extracts from the roots of *C. pilosula*, CPPs are well known for exhibiting strong immunomodulation activities [32]. In close agreement with the previous data, our study showed that the crude CPP content was highest on 5 September, which is consistent with the traditional harvest season (autumn) [33]. Although the monosaccharide composition assay showed results that were similar to those of a previous study [34], our study revealed a temperature-triggering carbon flux between primary and secondary metabolism, which was inferred from the dynamic changes in the contents of CPPs, soluble proteins, phenolics, and flavonoids.

Lobetyolin, an essential component of Dangshen, is garnering increasing attention for its significant pharmaceutical activities, such as its antitumor, antiviral, anti-inflammatory, mucosal protective, and antioxidant properties [8,35]. Several studies investigated the lobetyolin levels of Dangshen at different harvest periods; however, the trends were variable [19,36]. Our results indicated that the lobetyolin content in the sample collected on 5th June was highest and showed an apparent pattern of change: summer > autumn > winter (Figure 3A). The inconsistencies in the results implied that lobetyolin biosynthesis was regulated by multiple factors and is a complex process. Interestingly, the pattern of change in atractylenolide III was similar to that of lobetyolin. Although the lobetyolin and atractylenolide III contents were higher in summer, autumn was the more suitable harvest time considering the yield, which gradually increased from May to November.

Vitamin C has critical pleiotropic functions as an essential circulating antioxidant with anti-inflammatory and immune-supporting effects [37]. Here, we quantified the vitamin C and β-carotene contents of Dangshen. Unlike β-carotene, the dynamic changes in Vitamin C content showed a trend that was similar to that of the total antioxidant capacity of Dangshen.

The molecular biology of *C*. *pilosula* has attracted increasing attention in recent years. Candidate genes involved in polysaccharide biosynthesis have been identified based on their transcriptome sequences [38]. MeJA can promote the accumulation of bioactive metabolites by significantly upregulating the expression of the metabolite biosynthesis-related genes *CpUGPase* and *CpPMK* in C. *pilosula* [16]. Suitable reference genes for qRT-PCR under different experimental conditions have been confirmed [31], establishing a good foundation for accurately quantifying target gene expression levels. In this study, we focused on the correlation between the metabolite content and the candidate enzyme genes during different development stages and at different temperatures. Among these genes, we found that the expression level of *CpUGpase* was positively correlated with the CPP content, whereas *CpMVD* and *CpPMK* showed highly positive correlations with atractylenolide III (Figure 6). Increasing the expression levels of these key enzyme genes is a potential strategy for enhancing the production of bioactive compounds.

## 5. Conclusions

In the present study, we systematically analyzed the dynamic changes in the contents of multiple primary and secondary metabolites in annually harvested Dangshen from 20 May to 20 November 2020. Quantitative analysis of polysaccharides, soluble protein, and total phenolics and flavonoids provided insight into the carbon flux between primary and secondary metabolism in Dangshen. The dynamic changes in the contents of lobetyolin, atractylenolide III showed that the most suitable harvest time of Dangshen is autumn. The antioxidant capacity assay showed that the antioxidant capacity in Dangshen might be correlated with vitamin C levels. Correlation studies revealed a positive correlation between the CPP content and the expression level of *CpUGPase* and between the atractylenolide III content and *CpPMK* (*R*^2^ = 0.97) and *CpMVD* (*R*^2^ = 0.89) levels. These results might not only provide information for the harvest time of Dangshen but also lay a foundation for choosing cultivars with improved bioactive compounds using gene expression levels as a marker.

## Data Availability

All data analyzed during this study are included in the supplementary information files, and the DNA sequences have been deposited in the GenBank (https://www.ncbi.nlm.nih.gov/genbank/, accessed on 20 February 2023) under the accession number ON017518 (CpGAPDH), ON017519 (CpPMK), ON017520 (CpPMD), ON017521 (CpMVK), ON017522 (CpUGPase), ON017523 (CpUGE), and ON017524 (CpUGDH). The NCBI SRA IDs of *C. pilosula* are SRR10613471, SRR10613472, SRR10613473, SRR10613474, SRR10613475, SRR10613476, SRR10613477, SRR10613478, SRR10613479, and SRR10613480.

## Supplementary Materials

The following supporting information can be downloaded at: https://www.mdpi.com/article/10.3390/metabo13030456/s1, Table S1. Primer sequence used in this study for qRT-PCR; Figure S1: *Codonopsis pilosula* in the field; Figure S2. Root samples of *Codonopsis pilosula* at different developmental stages (scale bar, 5 cm); Figure S3: HPLC profiles of monosaccharide composition. Ms: Mixed standards; S: Sample; 1, D-mannose; 2, rhamnose; 3, D-glucuronic acid; 4, D-galacturonic acid; 5, glucose; 6, ga-lactose; 7, arabinose.
