# Supplementary material for: The Dynamic Changes in the Main Substances in Codonopsis pilosula Root Provide Insights into the Carbon Flux between Primary and Secondary Metabolism during Different Growth Stages
Source: Metabolites. 2023 Mar 21;13(3):456. doi: 10.3390/metabo13030456 (PMC10057730; doi:10.3390/metabo13030456)
Supplement: Supplementary file 1 [file metabolites-13-00456-s001.zip › metabolites-2233211-supplementary.pdf]

Table S1. Primer sequence used in this study for qRT-PCR

Gene	Primers
CpGAPDH	F: TGCTTCGTTCAACATCATTC<br>R: CATAACTGGCTGCCTTCTCC
CpPMK	F: TGCCGTAGTTGCTGCTTTACTT<br>R: TTCGTGGCTGTTTCTTGGTG
CpMVD	F: CAAGATGCTGGCGTTCAGG<br>R: CCTTTGGTTTTCTGCGTTGG
CpMVK	F:GACACAAAAGTTGGGAGGAACAC<br>R:GGTAGCCAGTTCATTGCTGATAGA
CpUGPase	F: TTTACCCTTGAGAACGACG<br>R: TCTGATGGCTATGTGACCC
CpUGE	F: CGGGGTACATCTGTGCTTG<br>R: ATGCCATACTTTGCCTTCC
CpUGDH	F: GATGCTTATGAGGCGACGAA<br>R:GAGGCTTACCAATGGAGTAGACAAT

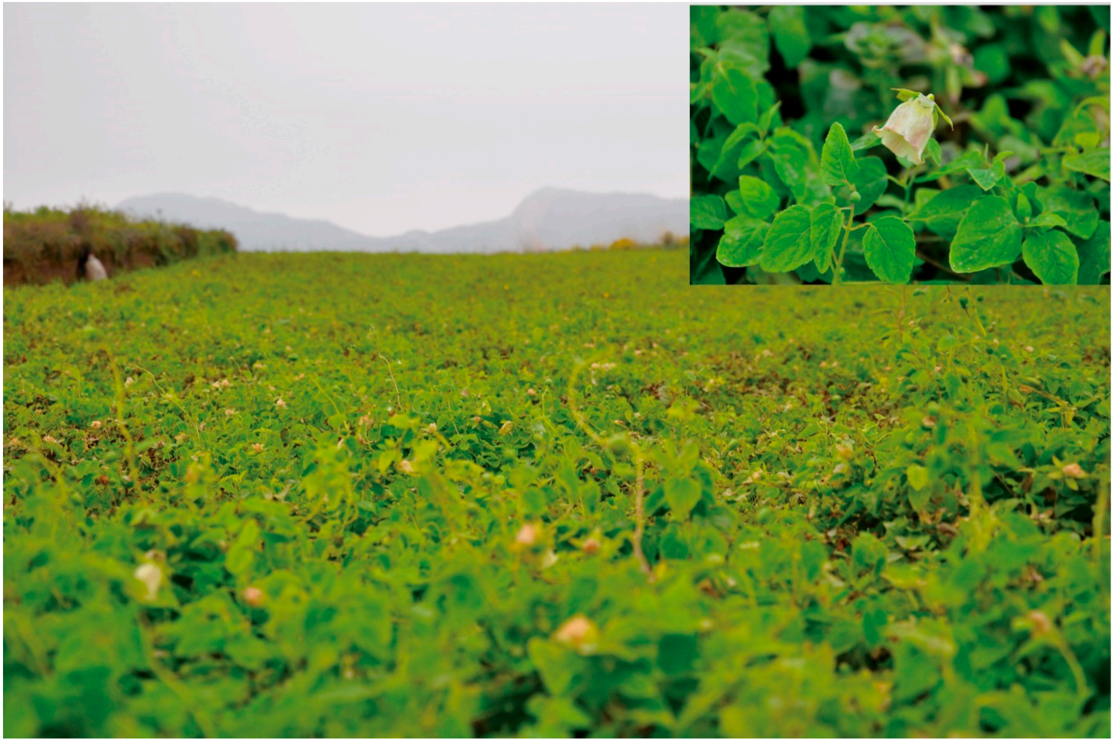

Figure S1. *Codonopsis pilosula* in the field.

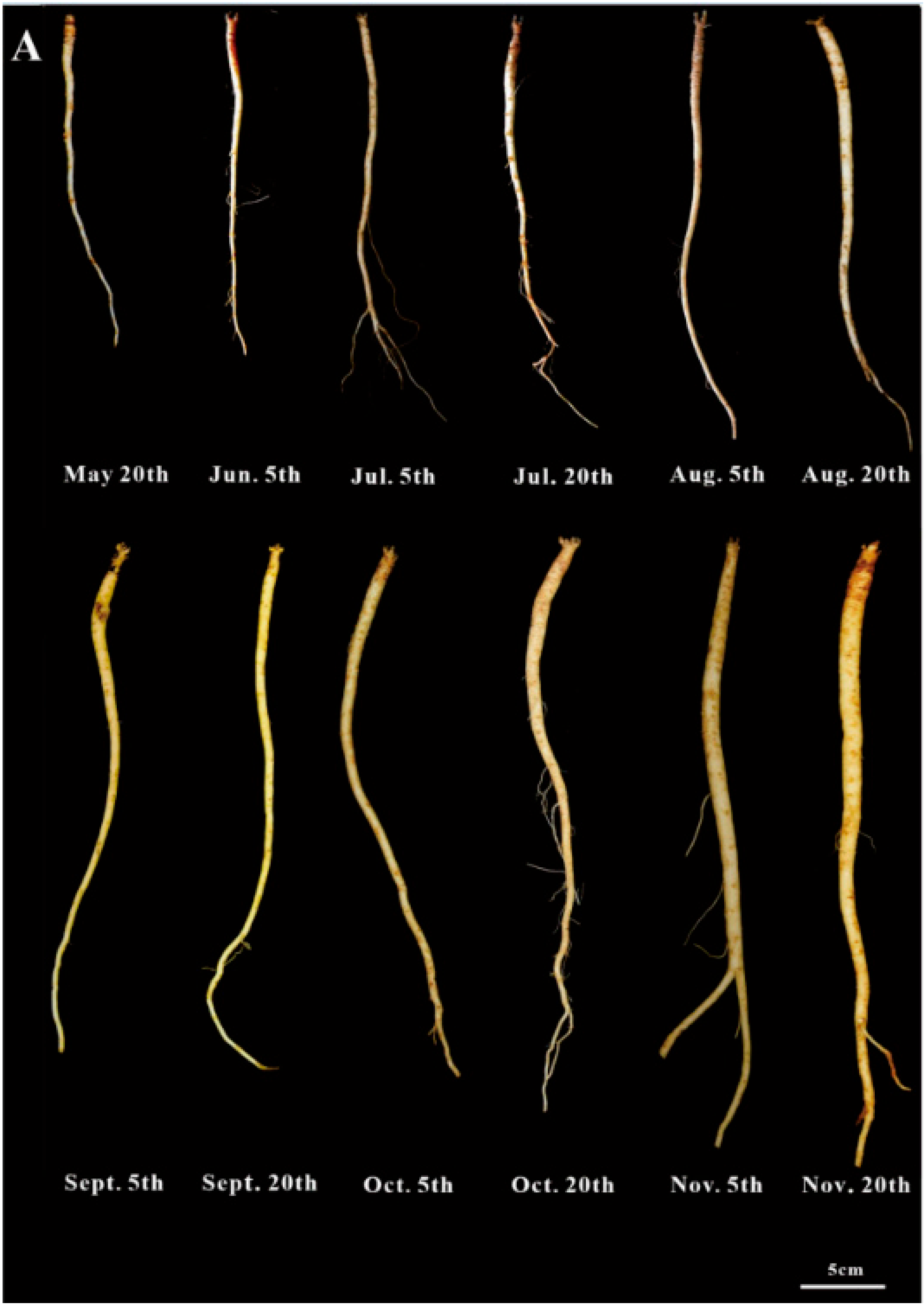

Figure S2. Root samples of *Codonopsis pilosula* at different developmental stages (scale bar, 5 cm)

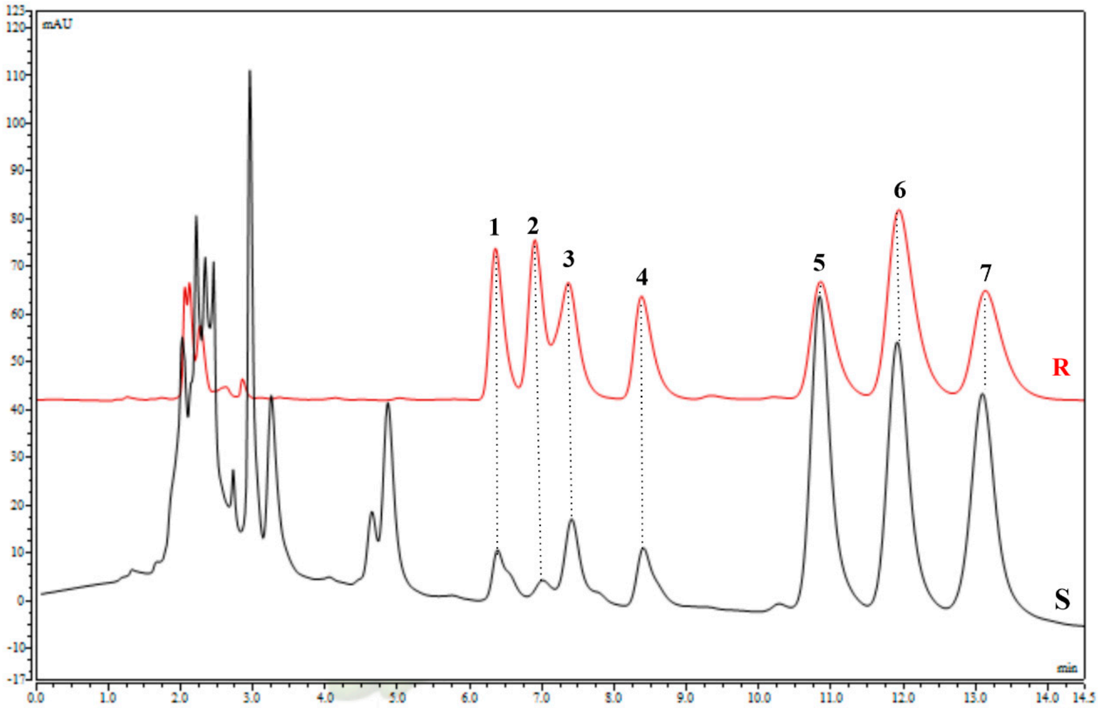

Figure S3. HPLC profiles of monosaccharide composition. Ms: Mixed standards; S: Sample; 1, D-mannose; 2, rhamnose; 3, D-glucuronic acid; 4, D-galacturonic acid; 5, glucose; 6, ga-lactose; 7, arabinose.
